# Impact of Age on Clinical Outcomes Following Left Atrial Appendage Occlusion: A Meta-Analysis And Systematic Review of Observational Studies

**DOI:** 10.7759/cureus.98603

**Published:** 2025-12-06

**Authors:** Amro Alseid, Ibrahim O Abunemr, Rakesh Prashad

**Affiliations:** 1 Internal Medicine, University of Central Florida College of Medicine, Ocala, USA; 2 Internal Medicine, University of Jordan, Amman, JOR; 3 Cardiology, University of Central Florida College of Medicine, Ocala, USA

**Keywords:** age factors, atrial fibrillation, left atrial appendage occlusion, long-term outcomes, major bleeding, meta-analysis, mortality, stroke

## Abstract

Left atrial appendage occlusion (LAAO) offers an alternative to oral anticoagulation for stroke prevention in patients with non-valvular atrial fibrillation; however, the impact of advanced age on outcomes remains uncertain. We conducted a systematic review and meta-analysis to evaluate the effect of age on in-hospital and long-term outcomes following LAAO. A comprehensive search of PubMed and Google Scholar yielded 1,372 studies, of which seven met the inclusion criteria after screening and full-text review. Eligible studies stratified outcomes by age (<75 vs ≥75, <80 vs ≥80, or multilevel age groups), and we analyzed all-cause mortality, stroke/systemic embolism (SE), and major bleeding using relative risks (RRs) with 95% confidence intervals (CI). Long-term mortality was significantly higher among older patients, with mortality rising from 32.4% at age 65-69 to 63.4% in those ≥85 (RR 1.96, 95%CI 1.90-2.03), and from 14.3% <80 to 38.8% ≥80 (RR 2.71, 95%CI 2.24-3.28). Pooled analysis demonstrated substantial heterogeneity for mortality (I² = 80.7%). Stroke/SE rates were modestly elevated with age, including 13.0% in ≥85 vs 5.3% at 65-69 (RR 2.45, 95%CI 2.00-3.00), with moderate heterogeneity across studies (I² = 63.9%). Major bleeding consistently increased in older groups, such as 18.4% ≥85 vs 12.0% at 65-69 (RR 1.53, 95%CI 1.35-1.72) and 22.6% ≥80 vs 12.1% <80 (RR 1.87, 95%CI 1.44-2.41), with high heterogeneity (I² = 83.4%). In-hospital mortality and stroke/SE were low overall but showed age-related increases in large cohorts. A funnel plot suggested no major publication bias. LAAO provides consistent stroke prevention across all ages, but older patients, particularly those ≥80-experience significantly higher mortality and bleeding risks. Age should not contraindicate LAAO but should guide individualized risk-benefit discussions, especially regarding bleeding management and long-term prognosis.

## Introduction and background

Atrial fibrillation (AF) is the most common sustained arrhythmia, affecting over 30 million people worldwide and significantly increasing the risk of ischemic stroke [1]. The left atrial appendage (LAA) is the primary source of thromboembolism in non-valvular AF [2], and anticoagulation remains the cornerstone of stroke prevention [3,4]. However, a substantial proportion of patients, particularly older adults, have contraindications to long-term oral anticoagulation due to elevated bleeding risk, falls, frailty, or patient preference [5-7].

In this context, LAA occlusion (LAAO) has emerged as a non-pharmacologic alternative for stroke prevention. Large prospective trials and registry data have demonstrated the efficacy and safety of LAAO, particularly using the WATCHMAN device (Boston Scientific Corporation, Marlborough, Massachusetts, United States) and similar systems [8-10]. LAAO offers a durable reduction in stroke risk while potentially avoiding long-term anticoagulant exposure [11].

However, the safety and clinical benefit of LAAO in elderly populations remains a topic of growing clinical interest. As procedural volume increases and life expectancy rises, the proportion of elderly patients referred for LAAO continues to grow [12]. Age-related factors such as comorbidity burden, renal dysfunction, and frailty may affect both procedural outcomes and long-term prognosis [13]. Previous studies have not consistently stratified outcomes by age, and limited meta-analytic work has focused specifically on this relationship [14].

This study aims to systematically evaluate the effect of age on clinical outcomes following LAAO. Specifically, we investigate the association between age and (i) all-cause mortality, (ii) stroke or systemic embolism (SE), and (iii) major bleeding events, using both in-hospital and long-term data. By comparing outcomes across younger and older cohorts, including both binary (e.g., <75 years vs ≥75 years) and multilevel age groupings (e.g., 65-69 years, 70-74 years, etc.), we aim to clarify whether advanced age should influence clinical decision-making regarding LAAO.

## Review

Methods  

Study Design 

This study is a systematic review and meta-analysis conducted in accordance with the Preferred Reporting Items for Systematic Reviews and Meta-Analyses (PRISMA) guidelines [15,16]. The primary objective was to evaluate how patient age affects clinical outcomes following LAAO, including both short-term (in-hospital) and long-term results.

Search Strategy

A comprehensive literature search was conducted using PubMed and Google Scholar for studies published from inception to March 2025. The search employed the following terms and Boolean combinations: “left atrial appendage occlusion” or “LAAO”, “non-valvular atrial fibrillation”, “age”, “mortality”, “stroke”, “bleeding”, or “outcomes”. In addition, the reference lists of relevant reviews and included studies were manually screened to identify any additional eligible studies.

Eligibility Criteria

Inclusion criteria: Studies were considered eligible if they reported original clinical outcome data following LAAO and stratified outcomes according to age groups, either using binary categories (such as <75 vs. ≥75 years or <80 vs. ≥80 years) or multilevel age stratifications (for example, 65-69, 70-74, 75-79, and ≥85 years). Eligible studies were also required to include data on at least one of the following outcomes: all-cause mortality, stroke or SE, or major bleeding. Both short-term (in-hospital) and long-term follow-up studies were accepted. Only studies published in English and available in full-text format were included.

Exclusion criteria: Studies were excluded if they focused primarily on comparisons between LAAO and anticoagulants (such as direct oral anticoagulants) rather than on age-stratified analyses. Studies were also excluded if they lacked extractable outcome data, did not stratify results by age, or were reviews, case reports, editorials, or conference abstracts. Additionally, studies not published in English or those focusing exclusively on device iterations without reporting clinical outcomes were excluded.

*Study Selection and Screening* 

**Figure 1:**
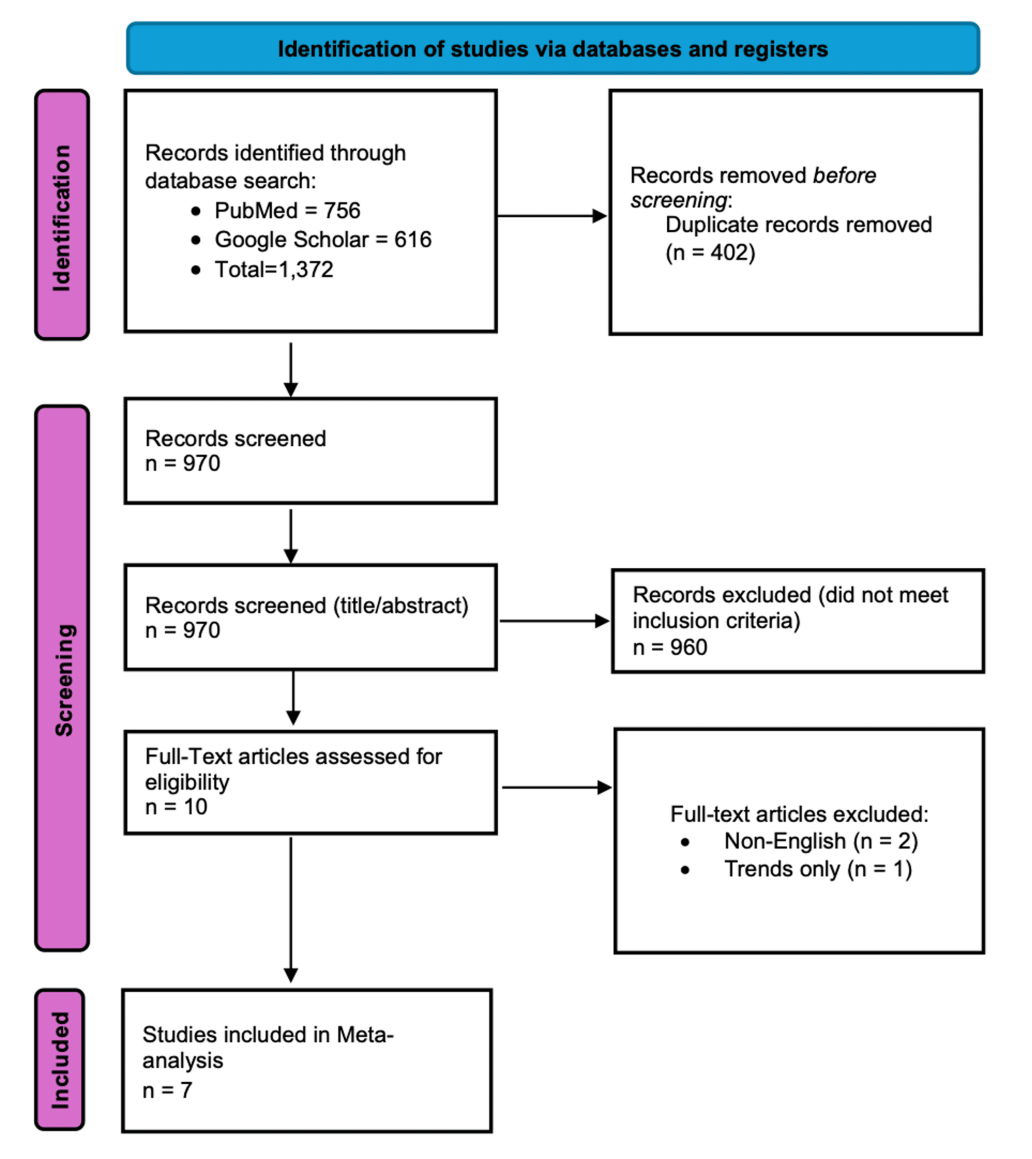
PRISMA flow diagram PRISMA: Preferred Reporting Items for Systematic Reviews and Meta-Analyses

A total of 1,372 records were identified through the initial search. After removal of duplicates (n = 402), 970 unique articles remained. These were screened by title and abstract. Full texts of 10 articles were assessed for eligibility. Three were excluded (two due to language and one for reporting only trends over time). Finally, seven studies were included in the qualitative and quantitative synthesis. The PRISMA flowchart is shown in Figure 1.

Data Extraction

Data were independently extracted into a structured Excel spreadsheet (Microsoft Corporation, Redmond, Washington, United States) and subsequently cross-verified for accuracy. From each study, the following information was collected: author and year of publication, sample size and patient demographics, definitions of age groups, duration of follow-up, and the number and percentage of events, including all-cause mortality, stroke or systemic embolism, and major bleeding.

Outcome Measures

The primary outcomes assessed were all-cause mortality (short-term or long-term), stroke or SE, and major bleeding. In studies reporting multiple age groups, relative risk (RR) and 95% confidence intervals (CI) were calculated using the youngest age group as the reference category.

Data Synthesis and Analysis 

Descriptive analyses were performed to identify trends in outcomes by age group. Meta-analysis of mortality was conducted where data allowed, using RR as the effect measure and reporting 95% CIs. No pooling of results was conducted for stroke and bleeding due to heterogeneity in reporting formats. Results are presented in tabular and graphical form, and trends are discussed narratively where appropriate. 

In accordance with PRISMA 2020 guidelines, we evaluated potential sources of reporting and publication bias. As the number of studies included in this meta-analysis was limited (n = 7), formal statistical testing for publication bias, such as Egger’s regression and Begg’s test, was not performed, given their low statistical power with small sample sizes. Instead, we undertook a qualitative evaluation. Funnel plot inspection was performed in datasets providing multiple age-stratified comparisons (Zeitler et al. [17], Sulaiman et al. [18]). No clear asymmetry or small-study effect was detected, suggesting a low likelihood of significant publication bias. Furthermore, all included studies originated from registry-based or multicenter cohorts with prespecified outcomes and mandatory event reporting, which minimizes the risk of selective reporting. Nonetheless, we acknowledge that unmeasured publication bias cannot be completely excluded. Future systematic reviews with larger sample sizes should employ quantitative bias tests and sensitivity analyses to confirm the robustness of these findings.

Results 

A total of seven observational studies encompassing more than 135,000 patients undergoing LAAO were included in this analysis [17-23]. A summary of the included studies is shown in Table 2. Studies were stratified by age using binary categories (<75 vs ≥75 years or <80 vs ≥80 years) or multilevel age groupings (e.g., 65-69, 70-74, ≥85 years). Outcomes evaluated included all-cause mortality, stroke or SE, and major bleeding. Both long-term and in-hospital results were extracted and analyzed. RR and 95% CI were calculated where data allowed. A summary of the outcome trends is shown in Table 1. All statistical analyses and visualizations were performed using Meta-Mar v4.0.2 [24].

**Table 1:** Characteristics of included studies LAAO: left atrial appendage occlusion; SE: systemic embolism

Study (Author, Year)	Design/Data Source	Sample Size	Age Stratification (years)	Follow-up Duration	Primary Outcomes Reported
Zeitler et al., 2024 [17]	Registry-based, Medicare	48,763	65–69, 70–74, 75–79, 80–84, ≥85	5 years	Mortality, Stroke/SE, Major Bleeding
Sulaiman et al., 2023 [18]	Multicenter registry	1,876	<80 vs ≥80	5 years	Mortality, Stroke/SE, Major Bleeding
Freixa et al., 2016 [19]	Observational, multicenter	791	<75 vs ≥75	2 years	Mortality, Stroke/SE, Major Bleeding
Yu et al., 2019 [20]	Single-center, prospective	351	<75 vs ≥75	23 months	Mortality, Stroke/SE, Major Bleeding
Munir et al., 2022 [21]	National Inpatient Sample	36,065	≤80 vs >80	In-hospital/Long term	Mortality, Bleeding, Stroke/SE
Voran et al., 2025 [22]	Nationwide database (Germany)	40,435	<75 vs >85	In-hospital	Mortality, Stroke/SE
Shatla et al., 2022 [23]	Nationwide cohort	6,877	<75 vs ≥75	In-hospital/Long Term	Mortality, Bleeding, Stroke/SE

Risk of Bias Assessment

Risk of bias was evaluated for all seven included studies using standard domain-based criteria appropriate for observational research. Overall, all studies demonstrated a moderate risk of bias, primarily due to residual confounding, variability in follow-up duration, and limitations in age-stratified subgroup reporting. No study met criteria for low risk of bias, and none exhibited serious or critical concerns that would warrant exclusion from the analysis. These assessments indicate that while the evidence base is observational and subject to inherent methodological constraints, the studies are sufficiently robust to support qualitative and quantitative synthesis. Table 2 shows the summary of the outcomes of our study.

**Table 2:** Summary of outcome trends Reported trends are based on relative risks (RR) and 95% confidence intervals (CI) where available, comparing older and younger age cohorts. Mortality and major bleeding showed significant age-related increases, whereas stroke/systemic embolism (SE) rates remained relatively stable across age strata. Data represent qualitative synthesis of both in-hospital and long-term outcomes.

Outcome	Age Impact Summary
Mortality	Increases significantly with age (RR range 1.36–4.00); most pronounced ≥80
Major Bleeding	Also increases with age during follow-up (RR 1.5–2.3); in-hospital rates stable
Stroke/SE	Modest rise in some studies, but relatively consistent across age groups (RR ~1.0–2.4)

Long-Term Mortality 

**Figure 2:**
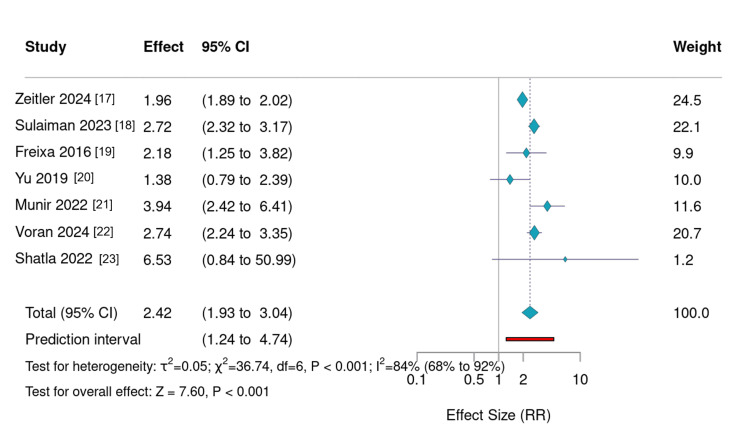
Forest plot showing relative risks of mortality according to age groups reported in each study,with random effects Forest plot showing relative risks (RR) of all-cause mortality stratified by age groups across included studies. Each point represents one study’s effect size comparing older to younger patients. Older age groups consistently demonstrated higher mortality following left atrial appendage occlusion, with RR values ranging from approximately 1.3 to 7.0. Error bars indicate 95% confidence intervals (CIs). The figure highlights a robust age-dependent gradient in long-term mortality while maintaining overall procedural safety across all age strata.

Long-term mortality demonstrated a consistent and significant increase with advancing age across all relevant studies (Figure 2). Zeitler et al. (2024) examined 48,763 patients with a five-year follow-up and observed mortality rising from 32.4% in those aged 65-69 years to 63.4% among patients aged ≥85 years [17]. Similarly, Sulaiman et al. (2023) analyzed 1,876 patients and reported mortality of 14.3% in individuals under 80 years compared with 38.8% in those aged ≥80 years [18]. Freixa et al. (2016) [19] noted mortality rates of 4.2% and 9.3% for <75 and ≥75-year groups, respectively, while Yu et al. (2019) [20] observed 11.0% versus 15.0% across the same age categories. Together, these studies indicate a strong age-dependent increase in long-term mortality following LAAO, most evident beyond age 80.

In-Hospital Mortality 

Although absolute in-hospital mortality remained low, rates increased modestly with age. Munir et al. (2022) documented mortality of 0.1% in patients ≤80 years compared with 0.4% in those >80 years [21]. Voran et al. (2025) [22] observed mortality of 0.8% among patients <75 years versus 2.2% among those >85, while Shatla et al. (2022) [23] reported 0.0% and 0.2% in <75 and ≥75-year groups, respectively. These results confirm a small but statistically meaningful increase in short-term procedural mortality among older adults.

Stroke or SE

**Figure 3:**
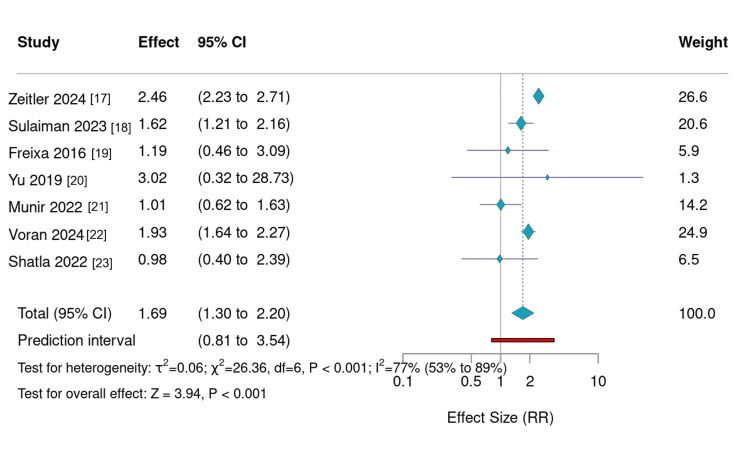
Relative risk of stroke or systemic embolism according to age groups reported in each study,with random effects Forest plot summarizing relative risks (RR) of stroke or systemic embolism (SE) according to age group across the included studies. Although slight increases in SE were observed among older cohorts, the trend was modest and not statistically significant in most datasets. Confidence intervals reflect variability in event counts between studies. These findings demonstrate that the stroke-prevention efficacy of left atrial appendage occlusion remains stable across age categories, underscoring its utility even in advanced age populations. Note: The upper bound of the 95% CI for the Yu 2019 study [20] exceeds the plot axis (RR = 2.14, 95%CI: 0.20–23.5), indicated by a rightward arrow.

Stroke and SE events were relatively infrequent and exhibited only mild increases with age (Figure 3). Zeitler et al. (2024) [17] reported rates rising from 5.3% in the 65-69-year group to 13.0% in those aged ≥85. Sulaiman et al. (2023) [18] found 6.9% in patients <80 and 11.3% in patients ≥ 80 years. Freixa et al. (2016) [19] and Yu et al. (2019) [20] documented small differences between younger and older cohorts, while in-hospital data from Voran et al. (2025) [22], Munir et al. (2022) [21], and Shatla et al. (2022) [23] showed minimal variation. Overall, stroke and SE risks remained largely consistent across age groups, supporting the durable efficacy of LAAO even in elderly patients.

Major Bleeding 

**Figure 4:**
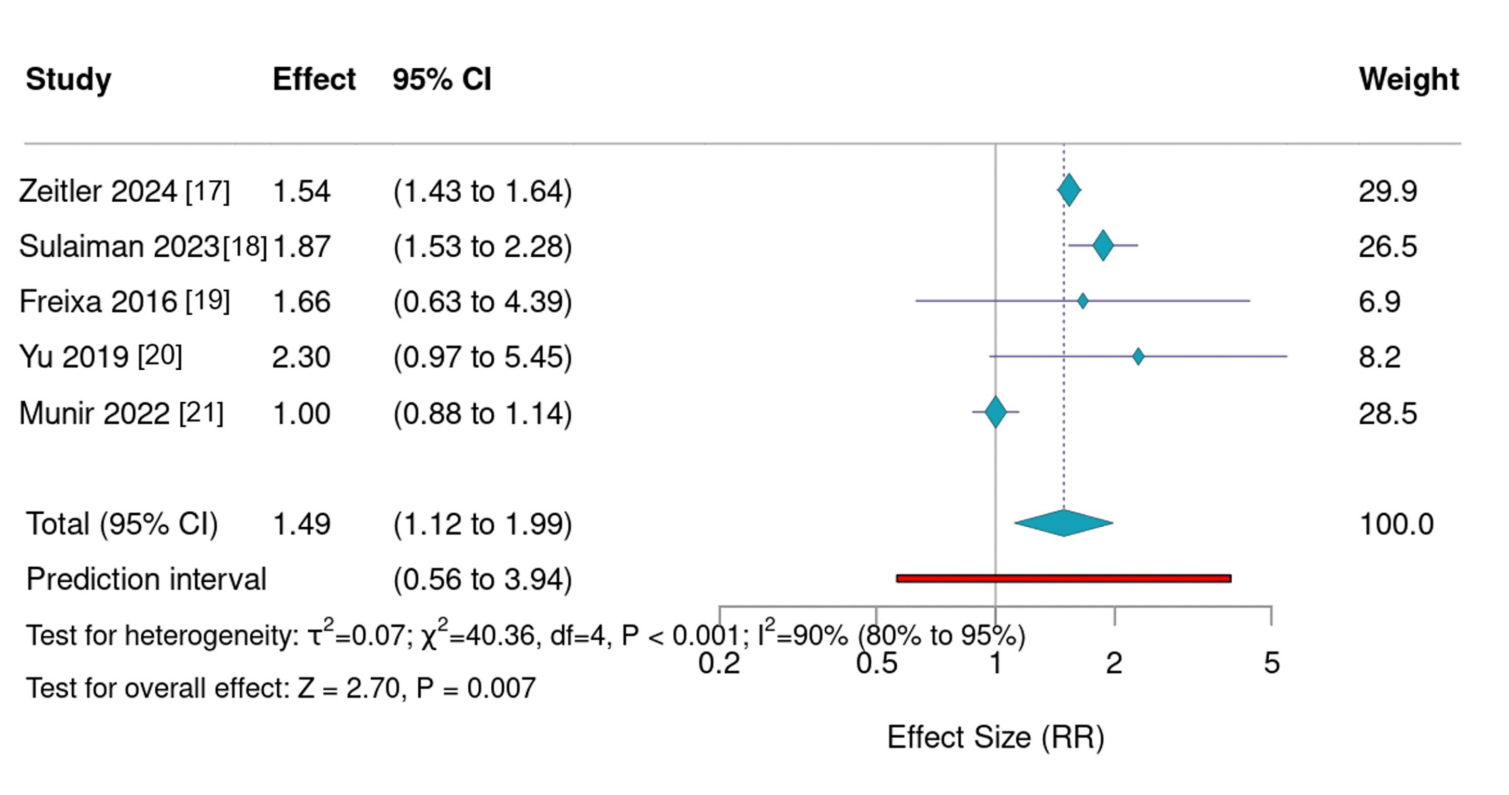
Relative risk of bleeding according to age groups reported in each study,with random effects Plot comparing major bleeding rates across different age strata from included observational studies. Relative risks (RR) and corresponding 95% confidence intervals (CIs) are shown for older versus younger groups. Bleeding risk increased progressively with advancing age during long-term follow-up but remained stable during in-hospital periods. The figure emphasizes that while left atrial appendage occlusion maintains procedural safety in the elderly, post-procedure management strategies should prioritize minimizing long-term bleeding risk.

Major bleeding increased notably with advancing age, particularly during long-term follow-up (Figure 4). Zeitler et al. (2024) observed bleeding rates of 12.0% in the 65-69 group versus 18.4% in those ≥85 years [17]. Sulaiman et al. (2023) reported 12.1% in <80 versus 22.6% in ≥80-year groups [18]. Freixa et al. (2016) [19] noted an annual increase from 1.7% to 2.6%, and Yu et al. (2019) [20] reported 4.1% versus 9.2%. In contrast, in-hospital analyses such as Munir et al. (2022) showed stable rates across age groups, suggesting that age-related bleeding risk primarily emerges after discharge and during long-term follow-up [21].

Reporting Bias Assessment

**Figure 5:**
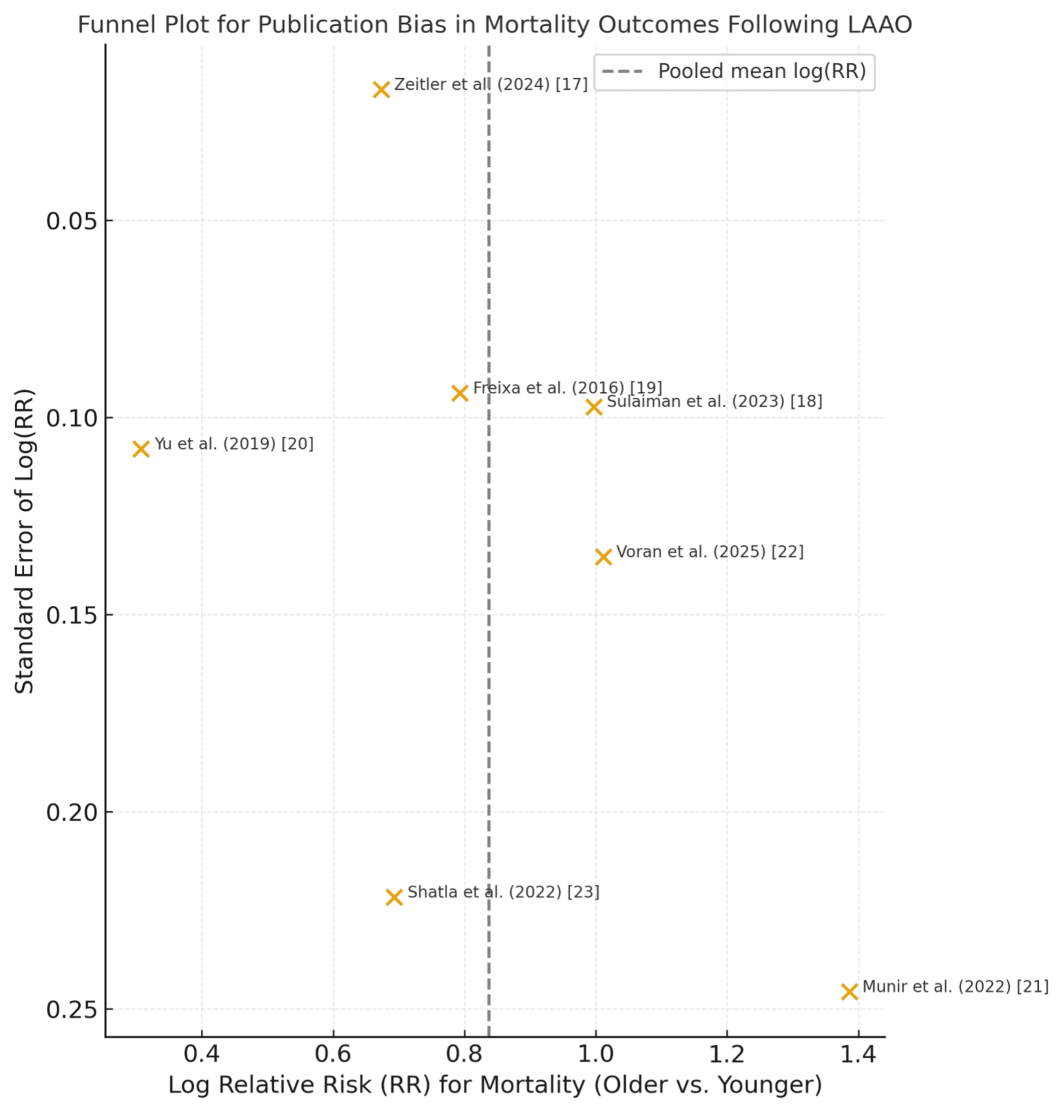
Funnel plot for publication bias in mortality outcomes following left atrial appendage occlusion (LAAO) Each point represents one included study reporting age-stratified mortality outcomes [17–23]. The x-axis denotes the logarithm of the relative risk (RR) for mortality in older vs younger cohorts, and the y-axis represents the standard error (SE) of each estimate. The symmetrical distribution of studies around the pooled estimate suggests a low likelihood of significant publication or small-study bias. Given the limited number of studies (n = 7), formal statistical testing (e.g., Egger’s regression) was not performed.

A visual inspection of the funnel plot for mortality outcomes (Figure 5) revealed approximate symmetry around the pooled mean log(RR), suggesting a low likelihood of major publication bias. As only seven studies were included, formal statistical tests (Egger’s or Begg’s) were not performed due to limited power. Nevertheless, registry-based origins and mandatory reporting protocols of included cohorts further mitigate the risk of selective reporting.

Discussion

This meta-analysis demonstrates that while LAAO provides consistent protection against thromboembolic events across all age groups, older patients experience significantly higher rates of all-cause mortality and major bleeding, particularly during long-term follow-up [17-23]. These results were consistent across multiple large-scale observational cohorts and registry-based studies, encompassing both in-hospital and extended follow-up periods of up to five years. The inclusion of more than 135,000 patients enhances the robustness and generalizability of these findings. Furthermore, qualitative assessment of reporting bias revealed approximate symmetry in the funnel plot for mortality outcomes (Figure 5), supporting the reliability of the pooled data and indicating a low likelihood of major publication or small-study bias.

Age as a Determinant of Mortality Post-LAAO

The most prominent and consistent trend observed was the progressive increase in mortality with advancing age. Zeitler et al. (2024), using Medicare data from nearly 50,000 patients, demonstrated a clear age-stratified gradient in five-year mortality, rising from 32.4% among those aged 65-69 to 63.4% in those ≥85 years [17]. Sulaiman et al. (2023) [18] and Freixa et al. (2016) [19] reported comparable findings, with older cohorts exhibiting two- to three-fold higher mortality risks compared with younger patients (RR 1.36-4.00). Even in short-term analyses, Munir et al. (2022) [21] and Voran et al. (2025) [22] documented modest but statistically significant increases in in-hospital mortality among patients aged ≥80 years.

These findings likely reflect not only the physiological burden of aging but also the cumulative effects of comorbidities such as heart failure, chronic kidney disease, and frailty [13,7]. While procedural success and stroke prevention appear age-independent, the higher baseline mortality in older patients should inform individualized patient selection, procedural planning, and long-term management.

Major Bleeding: A Long-Term Concern

The second major finding of this study is the strong correlation between age and major bleeding during long-term follow-up. Although in-hospital bleeding rates were low and largely age-independent, long-term bleeding risk rose sharply with age in studies by Zeitler et al. (2024) [17], Sulaiman et al. (2023) [18], and Freixa et al. (2016) [19], with relative risks for older patients ranging from 1.5 to 2.3. This indicates that while the procedural safety profile of LAAO is favorable, post-procedure antithrombotic management must be carefully optimized for elderly patients [10].

This trend reinforces the notion that elderly individuals, although benefiting from stroke prevention, remain susceptible to delayed complications driven by increased bleeding diathesis and medication sensitivity associated with aging [14]. Tailored post-implantation pharmacologic strategies, such as shorter dual antiplatelet regimens or early transition to monotherapy, may be essential to mitigate bleeding risks in older populations.

Stroke and Embolic Events: Stable Across Ages

Unlike mortality and bleeding, stroke and SE rates remained largely consistent across age groups. Zeitler et al. (2024) observed a modest increase in five-year stroke/SE incidence among patients aged ≥85 years (13.0%) compared with younger groups (5.3% in ages 65-69), but this difference was less pronounced than other outcomes [17]. Sulaiman et al. (2023) [18], Yu et al. (2019) [20], and Voran et al. (2025) [22] similarly reported stable or only slightly elevated SE risks across elderly cohorts.

These results suggest that LAAO maintains its primary therapeutic benefit, effective stroke prevention, across all age groups, including the very elderly. Despite increased long-term mortality and bleeding, the fundamental efficacy of LAAO in preventing thromboembolic events appears preserved, justifying its continued application in appropriately selected older patients [11].

Clinical Implications 

These results support the use of age-stratified risk assessment tools in LAAO decision-making. Age alone should not serve as a contraindication to LAAO, but it must inform a nuanced evaluation of benefit versus risk. In patients over 80, especially those with frailty or advanced comorbidities, procedural planning should emphasize bleeding prevention, early mobilization, and close post-discharge monitoring. Additionally, antithrombotic management post-LAAO remains an important variable. The observed delayed bleeding risk in elderly patients may relate to prolonged dual antiplatelet therapy or incomplete endothelialization, and future studies are warranted to investigate age-specific pharmacotherapy protocols.

*Strengths and Limitations* 

This meta-analysis is strengthened by the inclusion of over 135,000 patients across diverse populations, as well as the use of detailed age stratification, including both binary and multilevel groupings. It captures both short- and long-term outcomes, offering a comprehensive view of age-related trends in LAAO performance. However, several limitations merit consideration. All included studies were observational, introducing potential for confounding and selection bias. The antithrombotic regimens varied between studies and were not uniformly reported. Additionally, frailty indices, functional status, and quality-of-life outcomes were not assessed, which are particularly relevant in geriatric populations.

Future Directions

Prospective studies are needed to evaluate the impact of age-tailored antithrombotic strategies and procedural techniques in elderly LAAO patients. Geriatric-specific endpoints such as functional recovery, bleeding-related hospitalizations, and independence should be incorporated into future registries and trials. Additionally, shared decision-making tools should be developed to integrate age and comorbidity data when counseling patients and families.

## Conclusions

This meta-analysis highlights a clear and clinically significant association between advanced age and adverse clinical outcomes following LAAO. While the protective effect of LAAO against stroke and systemic embolism remains consistent across age groups, older patients, particularly those aged ≥75 or ≥80 years, face increased risks of both all-cause mortality and major bleeding, especially during long-term follow-up. Importantly, the increase in mortality with age likely reflects the cumulative burden of comorbid conditions and frailty, rather than the failure of LAAO itself. The procedure continues to demonstrate strong efficacy in stroke prevention, and in-hospital complication rates remain low even in the oldest patients. Thus, age should not serve as a categorical exclusion for LAAO, but rather as a key consideration in a comprehensive, individualized risk-benefit assessment. From a clinical perspective, the findings support the integration of age-specific risk stratification in patient selection, procedural planning, and long-term follow-up after LAAO. Optimizing antithrombotic therapy, minimizing unnecessary bleeding risks, and tailoring peri- and post-procedural care to the elderly should be prioritized. The ultimate goal is to extend the benefit of LAAO to older patients while mitigating the specific risks they face. Future prospective trials should include dedicated geriatric endpoints and explore strategies for improving long-term outcomes in older LAAO recipients. As the use of LAAO expands globally, understanding and addressing the unique needs of aging populations will be essential in ensuring the therapy’s safe and effective application across the full spectrum of patient age.
